# The Dentist’s Shooting Match

**Published:** 1860-02

**Authors:** 


### THE DENTIST'S SHOOTING MATCH.

The New York Journal is the only one that took notice of the shooting match at Niagara. We forgot all about it; but our readers who heard of it, of course, took it for granted that *Hunter* won the prize. *Western Hunters* do such things. But if, as the Journal tells us, "the shooting was very equal," on what principle was the award made? If the shooting were merely *equal* it would be difficult to determine who was victor, but when *very* equal it would be still more so. W.

